# Introduction to the Special Issue: Across the horizon: scale effects in global change research

**DOI:** 10.1093/aobpla/plv079

**Published:** 2015-07-14

**Authors:** Elise S. Gornish, Sebastian Leuzinger

**Affiliations:** 1 Plant Sciences, University of California, Davis, CA 95616, USA; 2 International Centre, Private Bag 92006, Auckland 1142, New Zealand

**Keywords:** CO_2_, community ecology, global change, spatiotemporal scale, warming

## Abstract

As a result of the increasing speed and magnitude in which habitats worldwide are experiencing environmental change, making accurate predictions of the effects of global change on ecosystems and the organisms that inhabit them have become an important goal for ecologists. Experimental and modelling approaches aimed at understanding the linkages between factors of global change and biotic responses have become numerous and increasingly complex in order to adequately capture the multifarious dynamics associated with these relationships. However, constrained by resources, experiments are often conducted at small spatiotemporal scales (e.g. looking at a plot of a few square metres over a few years) and at low organizational levels (looking at organisms rather than ecosystems) in spite of both theoretical and experimental work that suggests ecological dynamics across scales can be dissimilar. This phenomenon has been hypothesized to occur because the mechanisms that drive dynamics across scales differ. A good example is the effect of elevated CO_2_ on transpiration. While at the leaf level, transpiration can be reduced, at the stand level, transpiration can increase because leaf area per unit ground area increases. The reported net effect is then highly dependent on the spatiotemporal scale. This special issue considers the biological relevancy inherent in the patterns associated with the magnitude and type of response to changing environmental conditions, across scales. This collection of papers attempts to provide a comprehensive treatment of this phenomenon in order to help develop an understanding of the extent of, and mechanisms involved with, ecological response to global change.

## Abstract (precis)

A succinct introduction to the special issue focusing on scale effects in global change research.

## Introduction

Although research has already considered how spatial, organizational and temporal scales might modify conclusions about a variety of ecological dynamics including community assembly process (Suding *et al*. 2003; de Bello *et al*. 2013; Munkemüller *et al*. 2014), population growth rate (Villellas *et al*. 2013) and biological interactions (Wiens 1989; Strengbom *et al*. 2006; Riginos *et al*. 2009), the field of global change appears to be lagging behind in embracing the role of scale in mediating biotic response to a changing environment. This is problematic because the design and implementation of broad-scale management strategies to address conservation issues in the face of global change is often informed through extrapolating the more voluminous and detailed results of research conducted at local or small spatial scales to large scales (e.g. Hairston 1990; Cheruvelil *et al*. 2013), even though local-scale dynamics might not linearly translate to larger-scale patterns (Jarvis and McNaughton 1986; Donalson and Nisbet 1999; Tricker *et al*. 2009; Harte 2011; Supp *et al*. 2012). For example, at small scales (a single woodland), drought appears to be the major driver of treehole mosquito diversity and abundance, but at larger scales (across biogeographic regions), drought does not explain variation in mosquito number (Srivastava 2005). This is thought to occur because the mechanisms that drive dynamics across scales differ (e.g. Supp and Ernest 2014). Clearly, there is an urgent need to synthesize the complexity associated with over-arching trends that might be valid across large spatial, temporal and organizational dimensions. This special issue provides broad perspectives on global change research, dealing with plant response to environmental change across ecosystems, temporal and spatial scales, and organizational levels. It is our hope that this collective body of work will highlight trends in global change research that inform our understanding of how we affect our ecosystems.

## Contributions

**Niglas, Kupper, Tullus and Sellin** (2014) used a unique experimental design (the Free Air Humidity Manipulation facility) to investigate the effects of elevated atmospheric humidity on two levels of organization of hybrid aspen saplings growing in an experimental deciduous forest. They found that elevated air humidity resulted in relatively large increases in stomatal conductance and decreases in intrinsic water-use efficiency, due to changes in soil water potential and subsequent response of plant hormone ABA. Humidity effects on above-ground current year tree growth (measured as relative height increments), however, were relatively small. This work suggests that variation in atmospheric humidity at high latitudes may expose trees to higher dehydration risk during weather extremes.

By coalescing published accounts of how community response to global change drivers might impact ecosystem function and a simple 30 species modelling exercise, **Langley and Hungate** (2014) used a novel fusion of community and ecosystem ecology to explain short-term patterns observed in global change experiments. They argue that failure to account for plant community shifts in response to multiple environmental changes may cause earth system models to overestimate ecosystem response to elevated CO_2_ and nitrogen enrichment.

**Schymanski, Roderick and Sivapalan** (2014) used a model to explore potential differences between responses of vegetation to elevated atmospheric CO_2_ concentrations at different time scales when considering optimal adaptation. Instead of specifically testing a hypothesis, the authors attempted to raise awareness for implications of optimality hypotheses about potential medium to long-term responses of vegetation to elevated CO_2_. They highlight that considering time scales of adaptation for different vegetation properties facilitates differentiating between a short-term response to elevated CO_2_ (stomatal down-regulation) and a longer term response (increase in leaf area, plant abundance, and potentially, rooting depths).

Using plant species richness data from 809 WWF terrestrial ecoregions and MODIS-derived NPP estimates, **McBride, Cusens and Gillman** (2014) linked the fields of ecology and biogeography and modelled productivity data to analyse the relationship between species richness and productivity in relation to ecoregion size. They found a strong effect of scale on the productivity–species richness relationship within the global ecoregion dataset. Specifically, using large region size bins with existing data results in a stronger and clearer productivity–diversity relationship then using smaller region size bins. They further show that failure to consider scale at large sampling grains can lead to serious misunderstandings of fundamental diversity pattern responses to factors associated with global change.

**Miller** (2015) used a unique multi-year plant and elevation survey of coastal vegetation on a barrier island in the Gulf of Mexico to separate the effects of dune elevation and disturbance—as a result of increases in storm intensity and frequency—on the plant community across dune habitats. The analysis allowed him to detect a synergy between dunes and their vegetation, highlighting the dynamic link between geomorphology and the plant community. Understanding how plant communities respond to disturbance across dune habitats may be important for predicting future effects of climate change on coastal ecosystems.

**Gornish** (2014) conducted an exploratory analysis to assess the effects of elevated temperature and nitrogen deposition at different levels of spatial organization—at the leaf level, the plant level and the community level, in the presence and absence of experimental invasion in an old-field plant community in northern Florida. She found that the magnitude of response changed depending on the spatial scale, but the presence of invasion did not appear to affect the relationship between spatial scale and effect size.

**Fay, Newingham, Polley, Morgan, LeCain, Nowak and Smith** (2015) conducted a synthesis in order to understand how soil moisture and changes in community structure in response to enriched CO_2_ contribute to ecosystem productivity along precipitation gradients and soil types in rangeland systems. They found that correlations between above-ground biomass and dominant plant species were modified by soil type. This extends previous work that demonstrates how increasing CO_2_ can produce effects across spatial scales as well as across multiple levels of ecological organization.

Finally, **Leuzinger, Fatichi, Cusens, Niklaus and Körner** (2015) provide an interesting perspective on the difficulties of capturing hidden and underappreciated feedbacks in global change experiments. They qualitatively argue how those feedbacks might change at different spatiotemporal scales and use an example from a model sensitivity analysis to demonstrate the potential magnitude of this ‘island effect’. To conclude, the authors urge experimenters to be aware of the potential issue and provide some opportunities for explicitly considering the island effect in experiments.

This special issue provides an overview of innovative research approaches that are specifically designed to promote a deeper ecological understanding of the effects of human-induced global change by explicitly addressing linkages between broad considerations of life on earth to biologically relevant responses from other organizational levels and spatial scales. Budgetary and logistic restrictions always exist to force both modelling and experimental work into a limited spatiotemporal or organizational context. However, we think awareness of scaling issues and cautious interpretation of results can often lead to a much better understanding and therefore ability to project future global change impacts. In order to further develop a comprehensive understanding of scale effects on plant response to global change, we encourage researchers to more regularly integrate assessments of global change across scale in their work, evaluate the fit between global models and local causation and investigate local vs. global mitigation of global change impacts.

## Sources of Funding

E.S.G. was supported by the Department of Plant Sciences at the University of California, Davis.

## Contributions by the Authors

Both E.S.G. and S.L. developed ideas and contributed to the writing of the paper.

## Conflict of Interest Statement

None declared.
